# Drug-1,3,4-Thiadiazole Conjugates as Novel Mixed-Type Inhibitors of Acetylcholinesterase: Synthesis, Molecular Docking, Pharmacokinetics, and ADMET Evaluation

**DOI:** 10.3390/molecules24050860

**Published:** 2019-02-28

**Authors:** Rabail Ujan, Aamer Saeed, Pervaiz Ali Channar, Fayaz Ali Larik, Qamar Abbas, Mohamed F. Alajmi, Hesham R. El-Seedi, Mahboob Ali Rind, Mubashir Hassan, Hussain Raza, Sung-Yum Seo

**Affiliations:** 1 Dr. M.A. Kazi Institute of Chemistry, University of Sindh, Jamshoro 76080, Pakistan; rabailrabailnoor@gmail.com (R.U.); mahboobalirind@gmail.com (M.A.R.); 2 Department of Chemistry, Quaid-I-Azam University, Islamabad 45320, Pakistan; mrpervaiz@gmail.com (P.A.C.); fayazali@chem.qau.edu.pk (F.A.L.); 3 Department of Physiology, University of Sindh, Jamshoro 76080, Pakistan; qamar.abbas.qau@gmail.com; 4 Department of Pharmacognosy, College of Pharmacy, King Saud University, Riyadh 11451, Saudi Arabia; malajmii@ksu.edu.sa; 5 Pharmacognosy Group, Department of Medicinal Chemistry, Biomedical Center (BMC), Uppsala University, SE-751 23 Uppsala, Sweden; Hesham.El-Seedi@fkog.uu.se; 6 Department of Biological Sciences, College of Natural Sciences, Kongju National University, 56 Gongjudehak-Ro, Gongju, Chungnam 314-701, Korea; mubashirhassan_gcul@yahoo.com (M.H.); hussain_solangi@yahoo.com (H.R.); dnalove@kongju.ac.kr (S.-Y.S.)

**Keywords:** mixed-type AChE inhibitors, ADMET parameters, pharmacokinetics, drug-likeness, synthesis, antioxidant activity, molecular docking, 1,3,4-thiadiazole-drug

## Abstract

A small library of new drug-1,3,4-thiazidazole hybrid compounds (**3a**–**3i**) was synthesized, characterized, and assessed for their acetyl cholinesterase enzyme (AChE) inhibitory and free radical scavenging activities. The newly synthesized derivatives showed promising activities against AChE, especially compound **3b** (IC_50_ 18.1 ± 0.9 nM), which was the most promising molecule in the series, and was substantially more active than the reference drug (neostigmine methyl sulfate; IC_50_ 2186.5 ± 98.0 nM). Kinetic studies were performed to elucidate the mode of inhibition of the enzyme, and the compounds showed mixed-type mechanisms for inhibiting AChE. The *K*i of **3b** (0.0031 µM) indicates that it can be very effective, even at low concentrations. Compounds **3a**–**3i** all complied with Lipinski’s Rule of Five, and showed high drug-likeness scores. The pharmacokinetic parameters revealed notable lead-like properties with insignificant liver and skin-penetrating effects. The structure–activity relationship (SAR) analysis indicated π–π interactions with key amino acid residues related to Tyr124, Trp286, and Tyr341.

## 1. Introduction

The development of robust and novel drugs for the treatment of Alzheimer’s disease (AD) continues to be a complicated challenge for medicinal chemists and drug designers [1]. In the last two decades, pharmacologists have devoted substantial effort to designing effective medications for the treatment of neurodegenerative disorders. AD is responsible for substantial human mortality, and occurs in aged people. The nerve cells in the human brain communicate via sensory hormonal responses, and during progressive AD, the communication between the nerves is lost, and people can fail to recall their past or events in the recent past [2]. Cholinesterase inhibitors can have beneficial effects against AD.

**Figure 1:**
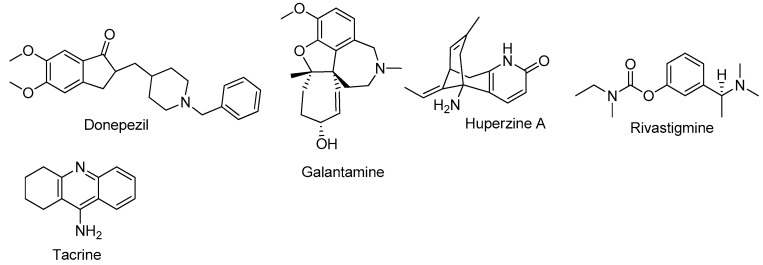
The acetyl cholinesterase enzyme (AChE) inhibitors currently employed in the treatment of Alzheimer’s disease (AD).

Acetyl cholinesterase (AChE, E.C. 3.1.1.7) is found in several types of tissues, including conducting tissues, peripheral tissues, and cholinergic and non-cholinergic tissues [3]. These enzymes hydrolyze acetylcholine (ACh), which is a neurotransmitter, into choline and acetic acid [4]. Thus, AChE inhibitors prevent the hydrolysis of ACh, maintaining the supply of this vital neurotransmitter in brain tissues to improve and stabilize the symptoms of dementia [5]. AChE terminates the signal pathway between the brain and nerve cells by effectively hydrolyzing ACh; a single molecule of AChE decomposes approximately 25,000 molecules per second [6,7]. The symptoms of AD are currently treated by exploiting the central cholinergic function of the FDA (Food and Drug Administration)-approved marketed drugs that are shown in Figure 1.

AChE inhibitors provide relief and improve the condition of patients suffering from AD by (a) improving their ability to think, (b) preventing memory loss, (c) and improving their behavioral and psychological conditions [8]. The long-term efficacy of prescribed drugs has been questioned over the years, and it depends on the response of the individual patient to the drug. In some cases, the drugs have remained beneficial for five years [9].

Sulfur-containing organic molecules have attracted special attention in the field of medicinal chemistry. Nitrogen and sulfur-containing heterocycles are commonly employed in the design of drugs in pharmaceutical chemistry [10]. The chemistry of 1,3,4-thiadizole dates back to 1882, when Fischer and Busch developed methods to synthesize its derivatives [11]. Since then, the chemistry of 1,3,4-thiadiazoles has expanded dramatically, and these fragments have been used in medicinal chemistry [12,13,14,15].

The coupling of two bioactive moieties has emerged as a promising strategy in drug design and discovery [16]. Herein, we report the hybridization of 1,3,4-thiadiazoles with various commercial carboxylic drugs to obtain single biologically active entities. The AChE inhibitory and free radical scavenging activities of these newly synthesized derivatives were evaluated. All of the new derivatives showed significant activity against AChE, and molecular docking studies elucidated the binding affinity of the target ligands into the active site of the target protein. Similarly, there are only a few reports of the free radical scavenging activities of such compounds. Therefore, it was envisioned to assess these compounds for their antioxidant potential, and two of the tested compounds exhibited exceptional radical scavenging potencies, which could signify the entry of a new class of antioxidants.

## 2. Experimental

### 2.1. General Procedure for the Synthesis of Drug-Derivatives 1,3,4-Thiadiazole (***3***)

An equimolar mixture of the drug (1.0 mmol) and thiosemicarbazide (1.0 mmol) in 3 mL of phosphoryl chloride was refluxed gently for 1 h. After completion of the reaction (according to TLC), the reaction mixture was brought to ambient temperature, and cold water (10 mL) was added. The resulting precipitate was isolated by filtration, washed with water, and recrystallized from ethanol to afford the amorphous target compounds.

*3-(5-amino-1,3,4-thiadiazol-2-yl)-1-cyclopropyl-6-fluoro-7-(piperazin-1-yl)quinolin-4(1H)-one* (***3a***). Yellow solid; yield: 78%, m.p: 225–227 °C; R_f_: 0.63 (Petroleum ether: ethyl acetate, 1:1); FTIR (neat, cm^−1^): 3135(C_sp2_-H), 1663 (C=O), 1589, 1541 (C=C, Ar), 1487 (N=O), 1251 (C=S); ^1^H-NMR (300 MHz, (CD_3_)_2_SO): δ (ppm) 12.67 (s, 1H, NH), 8.66 (s, 1H, ArH), 7.95 (s, 1H, ArH), 7.59 (s, 1H, C=CH), 6.09 (s, 1H, NH_2_), 3.85–3.79 (m, 1H, *C*H), 3.53–3.49 (m, 4H, CH_2_), 3.34–3.30 (m, 4H, CH_2_), 1.32 (d, 4H, CH_2_, *J* = 6.5 Hz); ^13^C-NMR (75 MHz, (CD_3_)_2_SO): δ (ppm) 179.1 (C=O, ketone), 166.2, 164.7, 148.6, 144.5, 139.5, 137.1, 122.3, 119.8, 119.7, 111.7, 111.4, 107.4, 46.9, 46.8, 43.1, 36.4, 8.1 Anal. Calcd. for C_18_H_19_FN_6_OS: C, 55.94; H, 4.96; N, 21.75; S, 8.30 found: C, 55.93; H, 4.92; N, 21.73; S, 8.31.

*(R)-6-(5-amino-1,3,4-thiadiazol-2-yl)-9-fluoro-3-methyl-10-(piperazin-1-yl)-2H-[1,4]oxazino[2,3,4-ij]quinolin-7(3H)-one* (**3b**). Pink solid; yield 82%, m.p. 247 °C (decomp.); [α]_20_^D^ + 103.7 (c 0.10, CH_3_OH)); IR (KBr) ν_max_: 1669 (C=O) and 3225 (N–H), ^1^H-NMR (MeOD, 300 MHz) δ: 8.51 (s, 1H, 5’aryl H), 6.34 (d, 1H, 8’aryl H, *J* = 13.1), 3.61; 2.59 (m, 8H, piperazinyl H), 4.31–4.22 (m, 3H, oxazine H), 3.65 (d, 3H, oxazine ring CH3, *J* = 6.1), 7.9 (s, NH_2_, amine), ^13^C-NMR (75 MHz, (CD_3_)_2_SO): δ (ppm) 182.0 (C=O, ketone), 162.95, 162.8, 159.8, 147.7, 143.6, 134.5, 128.1, 78.5, 66.5, 53.4, 49.5, 48.5, 18.7 Anal. Calcd. for C_19_H_21_FN_6_O_2_S: C, 54.79; H, 5.08; N, 20.18; S, 7.70 found: C, 54.81; H, 5.10; N, 20.21; S, 7.73.

*(R)-6-(5-amino-1,3,4-thiadiazol-2-yl)-9-fluoro-3-methyl-10-(4-methylpiperazin-1-yl)-2H-[1,4]oxazino[2,3,4-ij]quinolin-7(3H)-one* (**3c**). Light pink solid; yield 82%, m.p. 282 °C (decomp.); [α]_20_^D^–110.8 (c 0.10, CH_3_OH); IR (KBr) ν_max_: 1669 (C=O) and 3225 (N–H), ^1^H-NMR (MeOD, 300 MHz) δ: 8.51 (s, 1H, 5’aryl H), 6.34 (d, 1H, 8’aryl H, *J* = 13.1), 3.61; 2.59 (m, 8H, piperazinyl H), 2.34 (s, 3H, piperazinyl CH_3_), 4.31–4.22 (m, 3H, oxazine H), 3.65 (d, 3H, oxazine ring CH3, *J* = 6.1), 7.9 (s, NH_2_, amine), ^13^C-NMR (75 MHz, (CD_3_)_2_SO): δ (ppm) 1823.2 (C=O, ketone), 162.95, 162.8, 159.8, 147.7, 143.6, 134.5, 128.1, 78.5, 66.5, 53.4, 49.5, 48.5, 18.7 Anal. Calcd. for C_18_H_19_FN_6_O_2_S: C, 53.72; H, 4.76; S, 7.97 found: C, 53.01; H, 4.79; S, 7.93.

*5-(1-(4-isobutylphenyl)ethyl)-1,3,4-thiadiazol-2-amine* (**3d**). Off-white crystals; yield: 80%, m.p: 110 °C; R_f_: 0.52 (n-hexane: ethyl acetate 2:1); IR (KBr) (neat, cm^−1^): 3413, 3325 (N–H), 3143, 2956 (C_sp2_–H), 2823 (C_sp3_–H), 1598, 1443 (C=C, Ar), 1601 (C=N); ^1^H-NMR (300 MHz, DMSO-*d*_6_): δ (ppm) 7.20 (d, 2H, Ar–H), 7.12 (d, 2H, Ar–H), 7.00 (s, 2H, NH_2_), 4.35 (q, 1H, *J* = 7.14 Hz, CHCH_3_), 2.42 (d, 2H, *J* = 7.12 Hz, (CH_3_)_2_CHCH_2_Ar), 1.86 (m, 1H, CH(CH_3_)_2_), 1.59 (d, 3H, *J* = 7.2 Hz, ArCHCH_3_), 0.85 (d, 6H, *J* = 6.54 Hz, CH(CH_3_)_2_). ^13^C-NMR (75 MHz, (CD_3_)_2_SO): δ (ppm) 168.9 (C=N), 163.3, 141.4, 140.1, 129.6, 127.3, 44.6, 40.8, 30.0, 22.6, 21.4 Anal. Calcd. for C_14_H_19_N_3_S: C, 64.33; H, 7.33; N, 16.08; S, 12.27 found: C, 64.31; H, 7.35; N, 16.09; S, 12.26.

*(S)-5-(1-(6-methoxynaphthalen-2-yl)ethyl)-1,3,4-thiadiazol-2-amine* (**3e**). Dark brown crystals; yield: 85%, m.p: 118 °C; [α]_20_^D^–93.8 (c 0.10, DMSO); R_f_: 0.52 (n-hexane:ethyl acetate 2:1); IR (KBr) (cm^−1^): 3367–3182 (NH_2_); 3432–3250 (C–H); 1628–1607(C=N) cm^−1^. 1 H-NMR (DMSO-*d*_6_) 1.82 (d, *J* = 7.0 Hz, 3H, CH_3_); 3.91 (s, 3H, OCH_3_); 4.53 (q, *J* = 7.0 Hz, 1H, CH); 7.13–7.71 (m, 10H, ArH); 8.27 (s, 2H, NH_2_); ^13^C-NMR (126 MHz, CDCl_3_): δ 169.6, 161.9, 158.1, 134.0, 132.4, 130.8, 129.8, 129.4, 119.2, 105.7, 55.5, 45.1, 18.4. Anal. Calcd. for C_15_H_15_N_3_OS: C, 63.13; H, 5.30; N, 14.73; S, 11.24 found: C, 63.11; H, 5.32; N, 14.71; S, 11.24.

*5-((2-(4-((4-chlorophenyl)(phenyl)methyl)piperazin-1-yl)ethoxy)methyl)-1,3,4-thiadiazol-2-amine* (**3f**). White crystals; yield: 85%, m.p: 120 °C; R_f_: 0.52 (n-hexane:ethyl acetate 2:1); IR (KBr): 3413, 3325 (NH_2_), 1598, 1443 (C=C, Ar), 1601 (C=N); ^1^H-NMR (300 MHz, DMSO-*d*_6_): δ (ppm) 6.5 (s, 2H, NH_2_), 3.83 and 467 (m, 8H, piperazine), 3.84–3.91 (t, 2 × CH_2_), CH (s, 1H), 7.14–7.32 (m, ArH), ^13^C-NMR (75 MHz, (CD_3_)_2_SO): δ (ppm) 168.9 (C=N), 161.95, 143.8, 142.8, 131.7, 130.5, 130.6, 129.8, 129.5, 128.5, 126.5, 84.7, 71.7, 58.8, 54.6, 52.8. Anal. Calcd. for C_22_H_26_ClN_5_OS: C, 59.51; H, 5.90; N, 15.77; S, 7.22 found: C, 59.51; H, 5.92; N, 15.74; S, 7.20.

*3-(5-amino-1,3,4-thiadiazol-2-yl)-1-cyclopropyl-6-fluoro-7-(4-(3-nitrobenzoyl)piperazin-1-yl)qui nolin-4(1H)-one* (**3g**). Orange solid; yield: 76%, m.p: 225–227 °C; R_f_: 0.63 (petroleum ether:ethyl acetate,1:1); FTIR (neat, cm^−1^): 3262 (NH), 3135 (C_sp2_-H), 1663 (C=O), 1589, 1541 (C=C of Ar), 1487 (N=O), ^1^H-NMR (300 MHz, (DMSO-*d*_6_): δ (ppm) 8.82 (s, 1H, NH_2_), 8.80 (s, 1H, ArH), 8.39 (d, 1H, ArH, *J* = 8.8 Hz), 8.29 (s, 1H, ArH), 8.21 (d, 1H, ArH, *J* = 8.6 Hz), 7.93 (s, 1H, ArH), 7.71–7.67 (m, 1H, ArH), 7.51 (s, 1H, C=CH), 3.69–3.56 (m, 1H, CH), 3.19–3.15 (m, 4H, CH_2_), 2.64–2.59 (m, 4H, CH_2_), 1.11 (d, 4H, CH_2_, *J* = 6.8 Hz); ^13^C-NMR (75 MHz, (CD_3_)_2_ SO): δ (ppm) 191.0 (C=O of ketone), 158.5, 163.0, 169.3 (C=O, amide), 162.95, 151.1, 149.8, 146.7, 139.6, 134.5, 133.1, 132.6, 130.3, 129.4, 127.0, 114.6, 113.5, 51.7, 42.0, 34.6, 11.7. Anal. Calcd. for C_25_H_22_FN_7_O_4_S: C, 56.07; H, 4.14; N, 18.31; S, 5.99 found: C, 56.09; H, 4.16; N, 18.35; S, 5.97.

*3-(5-amino-1,3,4-thiadiazol-2-yl)-1-cyclopropyl-6-fluoro-7-(4-(4-methoxybenzoyl)piperazin-1-yl)quinolin-4(1H)-one* (**3h**). Yellow solid; yield: 82%, m.p: 172–175 °C; R_f_: 0.61 (petroleum ether: ethyl acetate, 1:1); FTIR (neat, cm^−1^): 3367 (NH_2_), 3010 (C_sp2_–H), 1669 (C=O), 1551, 1523 (C=C of Ar), 1090, 1250 (C–O); ^1^H-NMR (300 MHz, (DMSO-*d*_6_): δ (ppm) 7.82 (s, 1H, NH_2_), 8.73 (s, 1H, ArH), 7.95 (s, 1H, ArH), 7.81 (d, 2H, ArH, *J* = 7.5 Hz), 7.57 (s, 1H, C=CH), 7.05 (d, 2H, ArH, *J* = 7.5 Hz), 3.81 (s, 3H, OCH_3_), 3.59–3.54 (m, 1H, *C*H), 3.27–3.22 (m, 4H, CH_2_), 2.64–3.59 (m, 4H, CH_2_), 1.24 (d, 4H, CH_2_, *J* = 6.5 Hz); ^13^C-NMR (75 MHz, (CD_3_)_2_SO): δ (ppm) 193.0 (C=O of ketone), 160.5, 159.0, 167.3 (C=O of amide), 161.95, 151.8, 148.8, 145.7, 138.3, 134.5, 133.1, 131.6, 129.7, 128.3, 127.0, 115.6, 110.5, 61.2, 53.7, 45.0, 38.6, 10.7. Anal. Calcd. for C_26_H_25_FN_6_O_3_S: C, 59.99; H, 4.84; N, 16.14; S, 6.16 found: C, 59.96; H, 4.88; N, 16.17; S, 6.14.

*3-(5-amino-1,3,4-thiadiazol-2-yl)-1-cyclopropyl-7-(4-(2,4-dichlorobenzoyl)piperazin-1-yl)-6-fluoroquinolin-4(1H)-one* (**3i**). Pink solid; yield: 79%, m.p: 168–170 °C; R_f_: 0.61 (petroleum ether:ethyl acetate, 1:1); FTIR (neat, cm^−1^): 3263 (NH), 3090 (C_sp2_-H), 1667 (C=O), 1561, 1531 (C=C, Ar), 1265 (C=S), 735 (C-Cl); ^1^H-NMR (300 MHz, (DMSO-*d*_6_): δ (ppm) 7.02 (s, 1H, NH_2_), 8.80 (s, 1H, ArH), 8.29 (s, 1H, ArH), 7.77 (d, 1H, ArH, *J* = 8.9 Hz), 7.53 (s, 1H, ArH), 7.34 (d, 1H, ArH, 8.9 Hz), 7.28 (s, 1H, C=CH), 3.59–3.55 (m, 1H, *C*H), 3.29–3.26 (m, 4H, CH_2_), 2.54–2.49 (m, 4H, CH_2_), 1.21 (d, 4H, CH_2_, *J* = 6.3 Hz); ^13^C-NMR (75 MHz, (CD_3_)_2_SO):δ (ppm) 188.0 (C=O of ketone), 160.5, 159.0, 169.3 (C=O of amide), 162.95, 151.1, 149.8, 146.7, 139.6, 134.5, 133.1, 132.6, 130.3, 129.4, 127.0, 113.6, 111.5, 52.7, 41.0, 31.6, 10.7. Anal. Calcd. for C_25_H_21_Cl_2_FN_6_O_2_S: C, 53.67; H, 3.78; N, 15.02; S, 5.73 found: C, 53.64; H, 3.81; N, 15.01; S, 5.75.

#### Acetylcholinesterase Inhibition Assay

The inhibitory activities of the synthesized compounds were analyzed spectrophotometrically using acetylthiocholine iodide as the substrate by following the method of Ellman et al. [17]. In general, the reaction mixture comprised 180 µL of 50 mM of Tris HCl buffer (pH 8.0) with 0.1 M of sodium chloride and 0.02 M of magnesium chloride, to which 20 µL of enzyme (AChE, EC 3.1.1.7, AChE from human erythrocytes) solution was added (50 U in each well). The synthesized compounds (10 µL at the concentration being tested for their impact on growth) were added to the reaction mixture, and the mixtures were pre-incubated for 30 min at 4 °C. Then, 5,5′-dithiobis(2-nitrobenzoic acid) (0.3 mM, 20 µL) and acetylthiocholine iodide (1.8 mM, 20 µL) were added to the assay solution, and the mixtures were incubated at 37 °C for 10 min. After the incubation period, the absorbance of each well was measured at 412 nm. For the non-enzymatic reaction, the assays were carried out with a blank containing all the components except AChE. The assay measurements were collected at 475 nm using a microplate reader (OPTI Max, Tunable, Molecular Devices, Sunnyvale, CA, USA). IC_50_ values were calculated using GraphPad Prism 5.0 through non-linear regression. Each experiment was performed in triplicate.

The percentage of inhibition of tyrosinase was calculated: inhibition (%) = [(Blank−Sample)/Blank] × 100.

### 2.2. Kinetic Study

To determine the inhibition mechanism, a kinetic study was carried out. Compound **3b** was selected from the synthesized compounds for kinetic analysis to determine its inhibition potential. The identification of the inhibition mode of compound 3b began by using a series of concentrations of acetylthiocholine iodide (0.00 µM, 0.009 µM, 0.018 µM, and 0.036 µM) with various concentrations of **3b**. Briefly, acetylthiocholine iodide concentrations of 4 mM, 2 mM, 1, 0.5 mM, 0.25 mM, and 0.125 mM were used in the acetylthiocholine iodide kinetics studies, and the methods of all the kinetic studies were similar, as mentioned in the description of the AChE inhibition assay. The highest reaction rates were calculated from the initial linear portion of the absorbance plot, which represented the five minutes following the addition of the enzyme, and absorbance data were collected at 30-second intervals. The type of enzyme inhibition was determined from the Lineweaver–Burk plots of the inverse of velocity (1/V) versus the inverse of substrate concentration (1/[S] mM^−1^). The EI dissociation constant (*K*i) was determined from the secondary plot of 1/V versus inhibitor concentration.

### 2.3. Free Radical Scavenging Assay

The synthesized compounds were further evaluated for their 2,2-diphenyl-1-picrylhydrazyl (DPPH) radical scavenging capacity. To evaluate their DPPH inhibition abilities, a radical scavenging assay was performed [18,19]. The assay mixtures contained 20 µL of increasing concentrations of the test compounds and 100 µL of DPPH (150 µM), and the total volume of each well was brought up to 200 µL with methanol. Then, the mixture was incubated for 30 min at room temperature. For comparison and assay validity, ascorbic acid (vitamin C) was used as a positive control. The absorbance was recorded at 517 nm using a microplate reader (OPTI Max, Tunable). The results were calculated as percent inhibition. All of the concentrations were evaluated in triplicate.

### 2.4. Computational Methodology

#### Retrieval of the Protein Structure from the PDB

For computational analysis, the three-dimensional structure of human AChE (PDBID: 4PQE) was obtained from the Protein Data Bank (http://www.rcsb.org). Using the UCSF Chimera, a gradient algorithm and amber force field energy were minimized for further bioinformatics analysis. The 100 steepest descent steps with a step size of 0.02 Å were adjusted. Similarly, 10 conjugate gradient steps with a step size of 0.02 Å were also adjusted. The Discovery Studio 2.1 Client (D. Studio, 2008, BIOVIA, San Diego, CA, USA) was used to view the three-dimensional (3D) structure of the target protein. The Ramachandran graph of the protein was accessed through the Protein Data Bank (PDB). The basic structural protein architecture of helices, beta-sheets, coils, and turns was accessed by VADAR 1.8 (http://vadar.wishartlab.com/) [20]

### 2.5. Compound Structure

The prepared ligands (compounds **3a**–**3i**) were drawn in ACD/ChemSketch and minimized by UCSF Chimera 1.10.1. Compounds **3a**–**3i** were compared against Lipinski’s Rule of Five (RO5), and their biochemical applications were evaluated using online computational tools such as Molsoft (http://www.molsoft.com/) and Molinspiration (http://www.molinspiration.com/). Moreover, the pharmacokinetic properties, such as the absorption, distribution, metabolism, excretion, and toxicity (ADMET), of the synthesized compounds were evaluated through the pkCSM online server [20].

### 2.6. Molecular Docking

For the docking experiments, the PyRx docking tool was used for all the prepared compounds with the designated protein [21]. The grid box dimensions in the docking experiment were set as X =−25.27, Y = 22.43, and Z = 0.665 with a default exhaustiveness of eight. Each of the newly designed compounds was docked with the 3D structure of the target protein. Discovery studio and UCSF Chimera 1.10.1 were used for analysis of the docked complex through the lowest binding energy (Kcal/mol) and the hydrogen/hydrophobic interactions between the compounds and the amino acids in the protein. LIGPLOT was used to prepare two-dimensional (2D) graphical representations of the docked complexes [22,23].

## 3. Results and Discussion

**Scheme 1:**
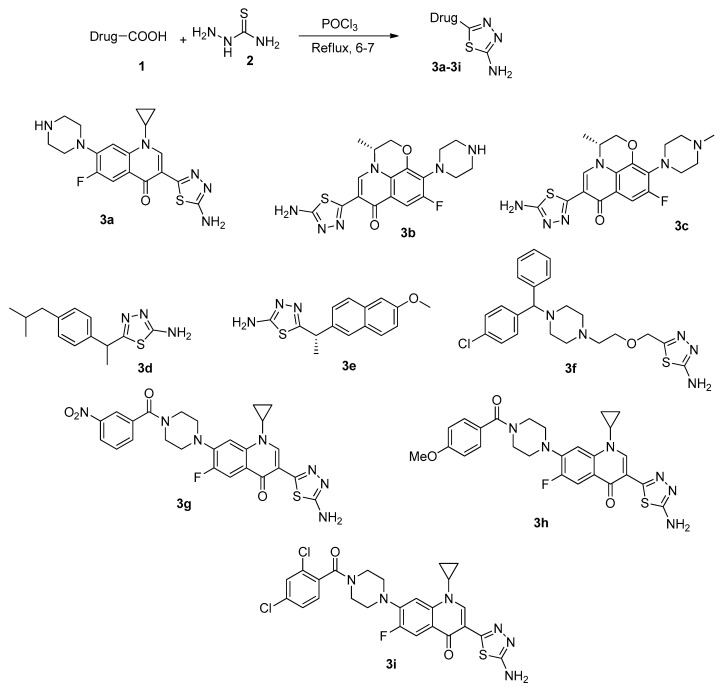
Synthesis of drug-like derivatives of 1,3,4-thiadiazoles (**3a–3i**).

The synthesis of the 5-amino-1,3,4-thiadiazole derivative drugs is depicted in Scheme 1. The carboxylic acid groups of the commercial drugs were cyclized onto thiosemicarbazide in dry ethanol to afford the desired products in good yields. The synthesized compounds were purified by recrystallization from aqueous ethanol.

The synthesized compounds were characterized by ^1^H-NMR and ^13^C-NMR spectroscopy. In their ^1^H-NMR spectra, the signals at approximately seven to eight ppm were assigned to the protons on the aromatic ring. The protons associated with the free amine moiety appeared between five and six ppm. In their ^13^C-NMR spectra, the sp^2^ carbons appeared between 100–140 ppm. The carbonyl groups resulted in the most deshielded signals in the spectra.

### 3.1. Acetyl Cholinesterase Inhibition Assay

The AChE inhibition studies of the synthesized compounds revealed that all the compounds selectively inhibited AChE in the nanomolar range (Table 1). (*R*)-6-(5-amino-1,3,4-thiadiazol-2-yl)-9-fluoro-3-methyl-10-(4-methylpiperazin-1-yl)-2*H*-[1,4]oxazino[2,3,4-ij]quinolin-7(3*H*)-one (**3b**), with an IC_50_ value of 18.1 ± 0.9 nM, was the most potent inhibitor of AChE. This compound showed substantially better activity than the reference drug, neostigmine methyl sulfate (IC_50_ 2186.5 ± 98.0 nM). Desmethyl levofloxacin **3b** lacks the 4-methyl group of piperazine moiety, which is similar in ciprofloxacin, but differs regarding the absence of cyclopropyl substituent compared with **3a**. Similarly, **3b** differs from levofloxacin **3c** in the absence of the 4-methyl group of piperazine moiety. Thus, presence of free NH in piperazine seems to play an important role in its activity. It may be attributed to the better orientation, conformational poses, and H-bonding interactions of **3b** with the active site of the enzyme. Comparative structure analysis indicated that compound **3a** also showed significant activity because of its cyclopropane ring, which was linked to the nitrogen atom. Compound **3i** showed poor activity relative to the other derivatives, because it possesses an acyl ring with two chloro substituents at the ortho and para positions with respect to the keto group. Compound **3f** moderately inhibited AChE, and it possessed an ether linkage and one chloro substituent on its aryl ring.

### 3.2. Kinetic Mechanism

**Figure 2:**
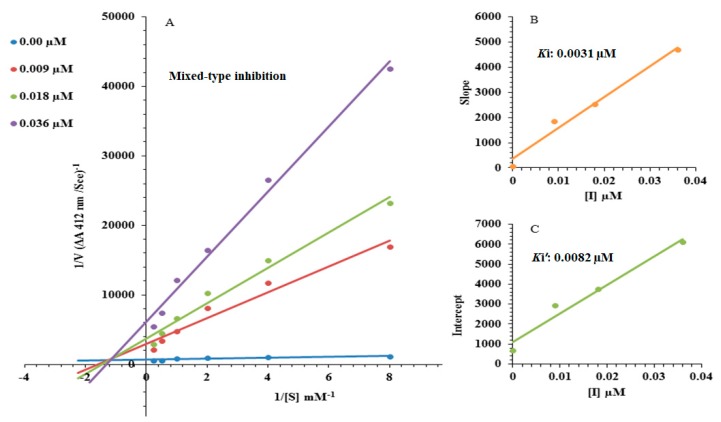
Lineweaver–Burk plots for the inhibition of AChE from human erythrocytes in the presence of compound **3b**. (**A**) The concentrations of **3b** were 0.00 µM, 0.009 µM, 0.018 µM, and 0.036 µM, and the substrate (urea) concentrations were 4 mM, 2 mM, 1 mM, 0.5 mM, 0.25 mM, and 0.125 mM. (**B**) The insets represent the plots of the slope (**C**) regarding the vertical intercepts versus the inhibitor concentrations, which were used to determine the inhibition constants.

Based on the IC_50_ values determined in this study, the most effective compound was **3b**; therefore, a kinetic study was carried out on **3b** to identify its mechanism of enzyme inhibition. The efficiency of **3b** in blocking the free enzyme and enzyme–substrate complex was investigated in terms of its enzyme inhibition (EI) and enzyme–substrate inhibition (ESI) constants. The inhibition of the enzyme was evaluated based on a Lineweaver–Burk plot of 1/V versus substrate (acetylthiocholine iodide) concentration (1/[S]) in the presence of various concentrations of inhibitor, and the linear plots are shown in Figure 2A. The plots of the effects of complex **3b** were linear and appeared in the second quadrant. The examination revealed that Vmax decreased with increasing K_m_ and increasing concentrations of the complex of **3b**, which indicated that this complex inhibits AChE in two distinct ways: competitively forming the EI complex and disrupting the enzyme–substrate–inhibitor (ESI) complex in a non-competitive manner. The graph of the slope versus the concentration of complex **3b** showed the EI dissociation constants (*K*i values) and is presented in Figure 2B; the ESI dissociation constants (*K*i′ values) are shown in the graph of intercept versus concentrations of complex **3b** in Figure 2C. *K*i was lower than *K*i′, indicating that the binding between the enzyme and **3b** was strong, suggesting ideal competitive behavior instead of non-competitive behaviour (Table 2). The kinetic constants and inhibition constants are presented in Table 2.

### 3.3. Free Radical Scavenging

**Figure 3:**
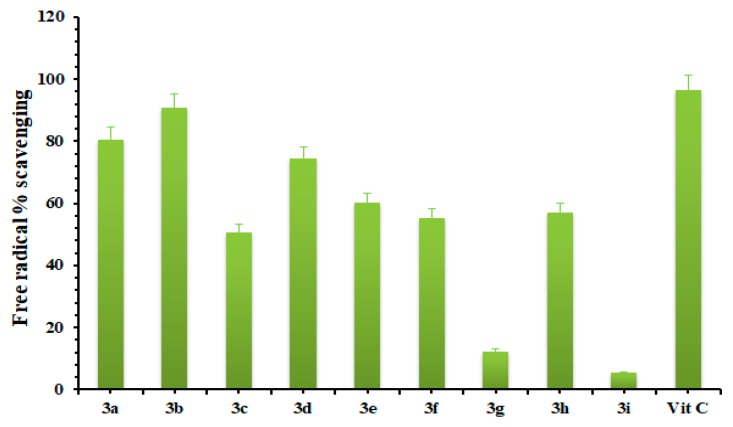
The percentage of free radical scavenging activities of the synthetic compounds presented as the mean ± SEM. All of the compounds were tested at a concentration of 100 µg/mL.

The newly prepared compounds were screened for their radical scavenging activities. Compounds **3a** and **3b** showed excellent radical scavenging potency in comparison to the reference drug vitamin C, while the other compounds did not show significant radical scavenging potency, even at high concentration (100 µg/mL). The better scavenging properties of **3a** and **3b** may be attributed to the presence of free piperazinic NH, which was not available in rest of the molecules. Meanwhile, the cyclopropane ring in **3a** decreases the activity. From the results discussed above, it may be concluded that the presence of free NH (similar to free OH in phenolics) is necessary for good antioxidant activities (Figure 3). 

### 3.4. Biochemical Properties and Lipinski’s Rule of Five (RO5) Validation

The biochemical applications of compounds **3a–3i** were predicted using computational tools (Molsoft and Molinspiration). The basic identified values are shown in Table 3. All of the prepared compounds were consistent with the RO5. The log *P* value and molecular mass should be less than five g/mol and 500 g/mol, respectively. Moreover, the compounds should have no more than 10 hydrogen bond acceptors (HBAs) and five hydrogen bond donors (HBDs). Being above the standards for HBAs and HBDs results in worse permeability [24], because hydrogen bonding has a substantial impact on permeability. Our results indicate that all the prepared compounds have <10 HBAs and <5 HBDs, making them consistent with the standard values. However, the log *P* values of all the prepared compounds were approximately equal to the standard value (>5). Multiple examples of existing drugs that violate with RO5 can be found [25,26,27].

### 3.5. ADMET Assessment of Synthesized Compounds

The physiological parameters, such as the absorption, distribution, metabolism, excretion, and toxicity (ADMET) of the present compounds were considered key hallmarks for identifying lead compounds [21]. The physiological properties of **3a–3i** are shown in Table 4. The absorption parameters, such as water solubility and intestinal solubility (percentage absorbed), overall absorption (log mol/L), and skin permeability (log K_p_), are indicators of the therapeutic efficacy of the synthesized complexes. The water solubility values for **3a**–**3i** were reasonable and revealed respectable absorption estimates. Additionally, **3a**–**3i** all showed respectable intestinal solubilities that were equivalent to the standard value (>30 %abs). The skin permeability values of the compounds were also approximately equal to the normal value (–2.5 log K_p_), which confirmed their drug-like properties. Additionally, the central nervous system (CNS) and blood–brain barrier (BBB) absorbency values of all the screened compounds were approximately equal to the normal values (>0.3 to <−1 log BB and >–2 to <–3 log PS) [21]. The results showed that these compounds were likely to cross these barriers and may be able to directly target the receptor molecules, which is of great importance. The anticipated toxicity and excretion values are also relevant to the drug-likeness behaviour of these compounds, and these parameters are evaluated on the basis of total clearance (log mL/min/kg), AMES toxicity, and maximum tolerated dose (MTD) and LD_50_ values [21]. The ADMET properties indicated that these novel compounds have acceptable lead-like potential with low hepatotoxic and no skin-sensitive effects.

### 3.6. Molecular Docking Analyses

**Figure 4:**
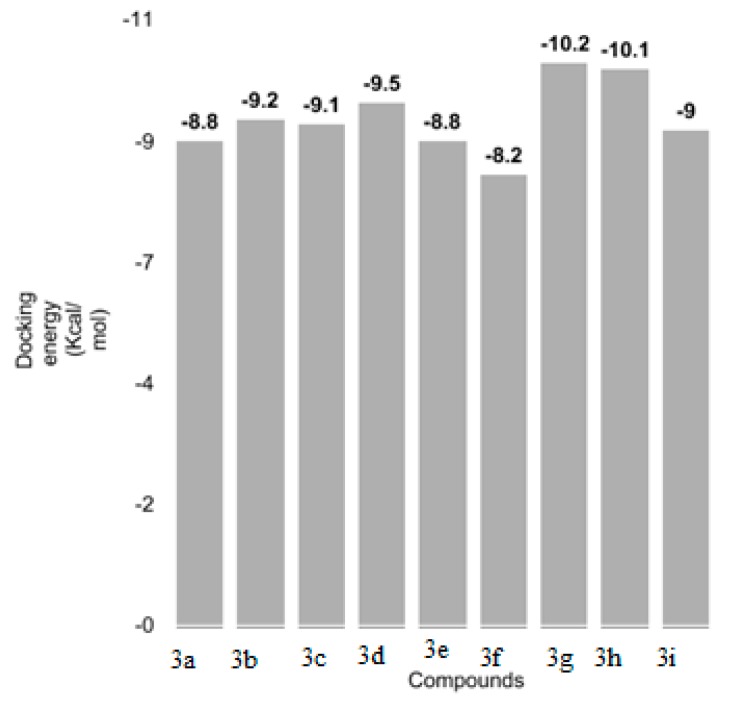
Docking energy values of all the synthesized docked compounds.

The docking of **3a**–**3i** was evaluated based on their hydrogen bonds, hydrophobic interactions, and lowest binding energy values (Kcal/mol) (Figure 4). The present findings indicated that **3g** and **3h** formed the best dynamic complexes, as they showed better binding energies (−10.20 and 10.10 Kcal/mol) than the other compounds. Additionally, the docked **3b** complex revealed a minimum energy of −8.20 Kcal/mol. The following equation was used to calculate the docking energies. The in vitro results showed that **3b** was the most active compared to other derivatives. However, the energy values in all the docking complexes were not fluctuated due to the common skeleton in all the compounds. The standard error of docking results for Autodock showed that the compounds with an energy difference greater than 2.5 Kcal/mol may be considered as good as any other form. However, in the present results, the deviated energy value is not greater than the standard value; therefore, the in vitro result of **5b** was the focus of the detailed interaction behavior in the active region of the target protein.

∆G binding = ∆Ggauss + ∆Grepulsion + ∆Ghbond + ∆Ghydrophobic + ∆Gtors(1)

Here, ∆Ggauss is an attractive term for the scattering of the two Gaussian functions, ∆Grepulsion: square of the distance if closer than a threshold value, ∆Ghbond: the ramp function also used for interactions with metal ions, ∆G hydrophobic: ramp function, ∆Gtors: contribution of the number of rotatable bonds.

### 3.7. Structure–Activity Relationship (SAR) Analyses between ***3b*** and Target Protein

**Figure 5:**
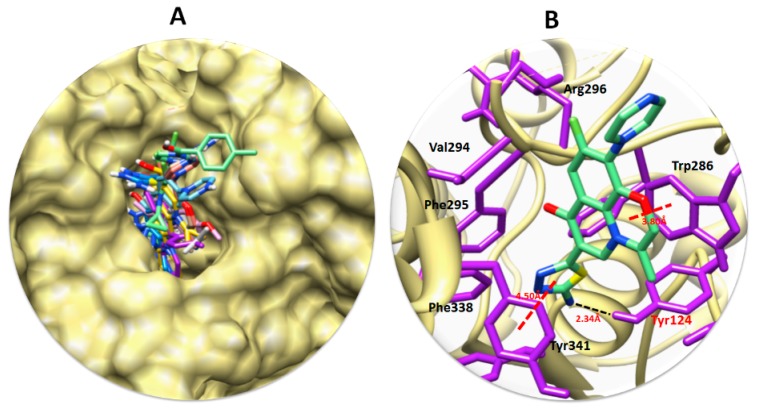
(**A**) Interactions of all the compounds within the active site of the target protein. (**B**) Docking interactions between **3f** and the target protein. **3f** is in light green, and the heteroatoms (oxygen, sulfur, and nitrogen) are shown in red, yellow, and blue, respectively. The protein is shown in khaki. The amino acids in the active site are highlighted in purple. The one hydrogen bond and two hydrophobic interactions are drawn in black and red lines, respectively.

All the synthesized compounds interact with the binding site in various conformations. Based on its in vitro IC_50_ and its in silico docking energy, **3b** was subjected to SAR analysis. Since **3b** showed the highest binding energy in the in silico study, it was nominated for evaluating different conformational poses in the target protein. The SAR analysis indicated that **3b** forms one hydrogen bond and two π–π interactions with Tyr124, Trp286, and Tyr341, respectively. The amino group of 3f interacts with Tyr124 and forms a strong hydrogen bond with a bond length of 2.34 Å. Likewise, two hydrophobic interactions were observed between Tyr341 and Trp286 with distances of 4.50 Å and 3.80 Å. A previous study reported that these cooperated residues are important in downstream signalling pathways [28,29]. A graphical depiction of the complex with 3f docked is shown in Figure 5. However, the binding pocket and all the other complexes with the prepared compounds docked are presented in the Supplementary Data (Figures S2–S9).

## 4. Conclusions

A series of 5-amino-1,3,4-thiadiazole derivatives were synthesized and assessed for their free radical scavenging and acetylcholinesterase (AChE) inhibitory activities. Compounds **3a**–**3i** all showed significant AChE inhibitory activities, with IC_50_ values in the nanomolar range. The most potent derivative, **3b**, was more active than the reference drug, neostigmine. Moreover, kinetic studies revealed a mixed mode of inhibition for the most potent derivative (**3b**). The ADMET parameters were evaluated to explore the pharmacokinetic profiles, and the experimental results (IC_50_) and values of the compounds showed appropriate correlation with the binding energy values (Kcal/mol). The Rule of Five (Lipinski’s rule) was also used to investigate the drug-likeness scores of derivatives **3a**–**3i**, and high drug-likeness scores were found. The molecular docking studies further elucidated the non-covalent interactions between the ligands and the active site of the target protein. In summary, further clinical trials and structural modifications may lead to the discovery of promising inhibitors of AChE, and could contribute to the treatment of Alzheimer’s disease (AD).

## Figures and Tables

**Table 1:** Acetylcholine esterase inhibitory activity of derivatives (**3a–3i**).

Compounds	Acetylcholine Esterase (from Human Erythrocytes) IC_50_ ± SEM (nM)
**3a**	126.3 ± 3.6
**3b**	18.1 ± 0.9
**3c**	576.3 ± 3.6
**3d**	2241.7 ± 112.0
**3e**	3806.4 ± 190.3
**3f**	17274.8 ± 863.0
**3g**	1182.19 ± 59.1
**3h**	1710.7 ± 86.5
**3i**	29228.0 ± 1461.4
Neostigmine methyl sulfate	2186.5 ± 98.0

Values are expressed as mean ± SEM; SEM = standard error of mean. Each experiment was performed in triplicate form.

**Table 2:** Kinetic parameters of the acetylcholine esterase from human erythrocytes for acetylthiocholine iodide activity in the presence of different concentrations of **3b.**

Concentration (µM)	V_max_ (ΔA/Sec)	K_m_ (mM)
0.00	0.001856	0.07692
0.009	0.000467	0.51282
0.018	0.000343	0.55555
0.036	0.000183	0.6060

V_max_ = the reaction velocity; K_m_ = Michaelis–Menten constant; *K*i = EI dissociation constant; *K*iʹ = ESI dissociation constant.

**Table 3:** Biological properties of synthesized compounds.

Properties	3a	3b	3c	3d	3e	3f	3g	3h	3i
Mol. weight (g/mol)	414	402	416	261	285	443	563	548	587
No. HBA	6	6	6	3	4	6	6	6	6
No. HBD	3	3	2	2	2	2	2	2	2
Mol. Log *P*	1.40	1.25	1.87	3.83	3.67	3.23	3.47	3.47	4.38
No of stereo centers	0	1	1	1	1	1	0	0	0
Mol. Vol (A^3^)	401	373	394	546	256	408	525	525	537
Drug likeness Score	0.90	0.40	0.97	0.85	0.50	2.46	0.94	1.05	0.90

**Table 4:** Pharmacokinetic assessment of synthesized compounds.

ADMET Properties		3a	3b	3c	3d	3e	3f	3g	3h	3i
Absorption	WS (log mol/L)	−3.031	−3.295	−3.814	−3.693	−2.925	−4.184	−3.903	−3.712	−3.766
	IS (%abs)	96.491	83.841	95.331	93.288	83.648	93.375	95.172	93.294	89.651
	SP (log K_p_)	−2.743	−2.815	−2.785	−2.741	−2.743	−2.895	−2.74	−2.741	−2.731
Distribution	BBBP (Log BB)	−1.267	−1.167	0.143	−0.926	−1.287	0.234	−1.261	−0.92	−1.114
	CNSP (Log PS)	−3.105	−3.134	−2.108	−3.346	−3.131	−2.087	−3.277	−3.346	−2.449
	VDss (log L/kg)	0.693	0.964	0.389	0.508	0.631	0.573	0.42	0.463	1.47
Metabolism	CYP3A4 inhibitor	No	No	No	Yes	No	No	Yes	Yes	Yes
	CYP1A2 inhibitor	No	No	Yes	No	Yes	Yes	No	No	No
	CYP2C19 inhibitor	No	No	Yes	Yes	No	Yes	Yes	Yes	No
	CYP2C9 inhibitor	No	No	Yes	Yes	No	No	Yes	Yes	No
Excretion	TC (log mL/min/kg)	0.508	0.871	0.097	0.009	0.585	−0.03	0.126	0.011	0.842
Toxicity	AMES toxicity	No	No	Yes	No	No	No	No	No	No
	Max. tolerat. dose	−0.245	−0.377	0.143	−0.07	−0.237	0.864	−0.057	−0.092	0.244
	ORAT(LD_50_)	2.528	2.924	2.914	2.605	2.483	2.669	2.593	2.606	2.698
	HT	Yes	Yes	No	Yes	Yes	No	Yes	Yes	Yes
	SS	No	No	No	No	No	No	No	No	No

Abbreviations: WS = water solubility, IS = intestinal solubility, SP = skin permeability, BBBP = blood–brain barrier permeability, CNSP = central nervous system permeability, TC = total clearance, ORAT = oral rat acute toxicity, HT = hepatotoxicity, SS = skin sensitization.

## Supplementary Materials

The supplementary materials are available online.

## References

[1] Fish P.V., Steadman D., Bayle E.D., Whiting P. (2019). New approaches for the treatment of Alzheimer’s disease. Bioorg. Med. Chem. Lett..

[2] Veitch D.P., Weiner M.W., Aisen P.S., Beckett L.A., Cairnsi N.J., Green R.C., Harvey D., Jack C.R., Jagustm W., Morris J.C. (2019). Understanding disease progression and improving Alzheimer’s disease clinical trials: Recent highlights from the Alzheimer’s Disease Neuroimaging Initiative. Alzheimer’s Dementia.

[3] Giacobini E. (2004). Cholinesterase inhibitors: New roles and therapeutic alternatives. Pharmacol. Res..

[4] Channar K.P.A., Shah M.S., Saeed A., Khan S., Larik F.A., Shabir G., Iqbal J. (2017). Synthesis, Characterization and Cholinesterase Inhibition Studies of New Arylidene Aminothiazolylethanone Derivatives. Med. Chem..

[5] Cummings J.L. (2000). Cholinesterase inhibitors: A new class of psychotropic compounds. Am. J. Psychiatry.

[6] Kavirajan H., Schneider L.S. (2007). Efficacy and adverse effects of cholinesterase inhibitors and memantine in vascular dementia: A meta-analysis of randomised controlled trials. Lancet Neurol..

[7] Saeed A., Shah M.S., Larik F.A., Khan S.U., Channar P.A., Flörke U., Iqbal J. (2017). Synthesis, computational studies and biological evaluation of new 1-acetyl-3-aryl thiourea derivatives as potent cholinesterase inhibitors. Med. Chem. Res..

[8] Torre P.D.L., Saavedra L.A., Caballero J., Quiroga J., Alzate-Morales J.H., Cabrera M.G., Trilleras J. (2012). A novel class of selective acetylcholinesterase inhibitors: Synthesis and evaluation of (*E*)-2-(benzo [d] thiazol-2-yl)-3-heteroarylacrylonitriles. Molecules.

[9] Munoz-Ruiz M.P., Rubio L., García-Palomero E., Dorronsoro I., del Monte-Millán M., Valenzuela R., Usán P., de Austria C., Bartolini M., Andrisano V. (2005). Design, synthesis, and biological evaluation of dual binding site acetylcholinesterase inhibitors: New disease-modifying agents for Alzheimer’s disease. J. Med. Chem..

[10] Terzioglu N., Gürsoy A. (2003). Synthesis and anticancer evaluation of some new hydrazone derivatives of 2, 6-dimethylimidazo [2,1-b][1,3,4] thiadiazole-5-carbohydrazide. Eur. J. Med. Chem..

[11] Clerici F., Pocar D., Guido M., Loche A., Perlini V., Brufani M. (2001). Synthesis of 2-amino-5-sulfanyl-1, 3, 4-thiadiazole derivatives and evaluation of their antidepressant and anxiolytic activity. J. Med. Chem..

[12] Schenone S., Brullo C., Bruno O., Bondavalli F., Ranise A., Filippelli W., Rinaldi B., Capuano A., Falcone G. (2006). New 1,3,4-thiadiazole derivatives endowed with analgesic and anti-inflammatory activities. Bioorg. Med. Chem..

[13] Oruç E.E., Rollas S., Kandemirli F., Shvets N., Dimoglo A.S. (2004). 1,3,4-thiadiazole derivatives. Synthesis, structure elucidation, and structure− antituberculosis activity relationship investigation. J. Med. Chem..

[14] Mavrova A.T., Wesselinova D., Tsenov Y.A., Denkova P. (2009). Synthesis, cytotoxicity and effects of some 1,2,4-triazole and 1,3,4-thiadiazole derivatives on immunocompetent cells. Eur. J. Med. Chem..

[15] Hu Y., Li C.Y., Wang X.M., Yang Y.H., Zhu H.L. (2014). 1,3,4-Thiadiazole: Synthesis, reactions, and applications in medicinal, agricultural, and materials chemistry. Chem. Rev..

[16] Pisani L., Farina R., Catto M., Iacobazzi R.M., Nicolotti O., Cellamare S., Mangiatordi G.F., Denora N., Soto-Otero R., Siragusa L. (2016). Exploring Basic Tail Modifications of Coumarin-Based Dual Acetylcholinesterase-Monoamine Oxidase B Inhibitors: Identification of Water-Soluble, Brain-Permeant Neuroprotective Multitarget. J. Med. Chem..

[17] Ellman G.L., Courtney K.D., Andres V., Featherstone R.M. (1961). A new and rapid colorimetric determination of acetylcholinesterase activity. Biochem. Pharmacol..

[18] Larik F.A., Saeed A., Channar P.A., Ismail H., Dilshad E., Mirza B. (2016). New 1-octanoyl-3-aryl thiourea derivatives: Solvent-free synthesis, characterization and multi-target biological activities. Bangladesh J. Pharmacol..

[19] Pettersen E.F., Goddard T.D., Huang C.C., Couch G.S., Greenblatt D.M., Meng E.C., Ferrin T.E. (2004). UCSF Chimera—A visualization system for exploratory research and analysis. J. Comput. Chem..

[20] Willard L., Ranjan A., Zhang H., Monzavi H., Boyko R.F., Sykes B.D., Wishart D.S. (2003). VADAR: A web server for quantitative evaluation of protein structure quality. Nucleic. Acids. Res..

[21] Pires D.E., Blundell T.L., Ascher D.B. (2015). pkCSM: Predicting small-molecule pharmacokinetic and toxicity properties using graph-based signatures. J. Med. Chem..

[22] Dallakyan S., Olson A.J. (2015). Small-molecule library screening by docking with PyRx. Methods Mol. Biol..

[23] Wallace A.C., Laskowski R.A., Thornton J.M. (1996). LIGPLOT: A program to generate schematic diagrams of protein-ligand interactions. Protein Eng..

[24] Studio D. (2008). Discovery.“Version 2.1.”.

[25] Kadam R.U., Roy N. (2007). Recent trends in drug-likeness prediction: A comprehensive review of in silico methods. Indian J. Pharm. Sci..

[26] Bakht M.A., Yar M.S., Abdel-Hamid S.G., Al Qasoumi S.I., Samad A. (2010). Molecular properties prediction, synthesis and antimicrobial activity of some newer oxadiazole derivatives. Eur. J. Med. Chem..

[27] Tian S., Wang J., Li Y., Li D., Xu L., Hou T. (2015). The application of in silico drug-likeness predictions in pharmaceutical research. Adv. Drug. Deliv. Rev..

[28] Fang J., Wu P., Yang R., Gao L., Li C., Wang D., Wu S., Du A.L., Liu G.H. (2014). Inhibition of acetylcholinesterase by two genistein derivatives: Kinetic analysis, molecular docking and molecular dynamics simulation. Acta Pharm. Sin. B.

[29] Simeon S., Anuwongcharoen N., Shoombuatong W., Malik A.A., Prachayasittikul V., Wikberg J.E., Nantasenamat C. (2016). Probing the origins of human acetylcholinesterase inhibition via QSAR modeling and molecular docking. PeerJ.

