# Supplementary material for: Drug-1,3,4-Thiadiazole Conjugates as Novel Mixed-Type Inhibitors of Acetylcholinesterase: Synthesis, Molecular Docking, Pharmacokinetics, and ADMET Evaluation
Source: Molecules. 2019 Feb 28;24(5):860. doi: 10.3390/molecules24050860 (PMC6429202; doi:10.3390/molecules24050860)
Supplement: Supplementary file 1 [file molecules-24-00860-s001.pdf]

Synthesis of drug-derivatives of 1,3,4-thiadiazole as novel mixed type inhibitors of acetylcholinesterase, and their molecular docking studies, Pharmacokinetics, ADMET parameters evaluation

Rabail Ujal, Aamer Saeed*a, Pervaiz Ali Channara Fayaz Ali Larika, Qamar Abbasb, Hesham R. El-Seedie, Mahboob Ali Rindd Mubashir Hassanc, Hussain Razac, Sung-Yum Seoc

[a] Department of Chemistry, Quaid-I-Azam University, Islamabad 45320, Pakistan.
[b] Department of Physiology, University of Sindh, Jamshoro76080, Pakistan
[c] Department of Biological Sciences, College of Natural Sciences, Kongju National University, 56 Gongjudehak-Ro, Gongju, Chungnam 314-701, Republic of Korea
[d] Dr. M. A. Kazi Institute of Chemistry, University of Sindh, Jamshoro, Pakistan
[e] Bio-Medical Center (BMC), Medicinal Chemistry, Division of Pharmacognosy, Department of Medicinal Chemistry, Uppsala University, Biomedical Centre, Uppsala, SE Box 574, SE-75

Correspondence: aamersaeed@yahoo.com, Tel +92-51-9064-2128; Fax: +92-51-9064-2241

* Email: aamersaeed@yahoo.com,/fayazali@chem.qau.edu.pk Tel +92-51-9064-2128; Fax: +92-51-9064-2241

Supplementary Information File

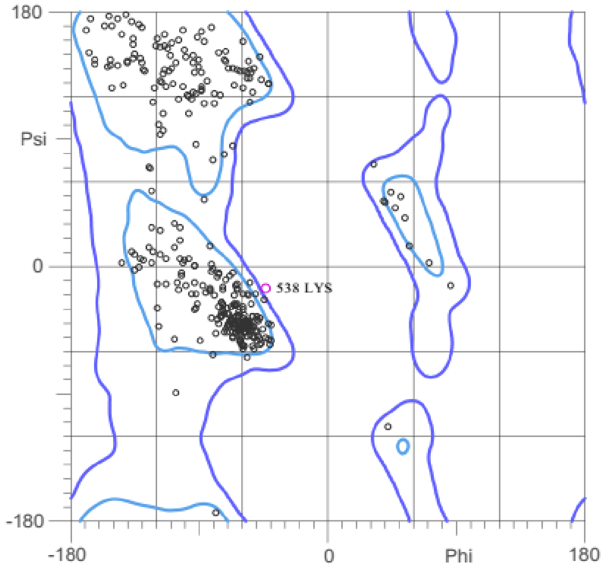

Figure. S1 Ramachandran graph.

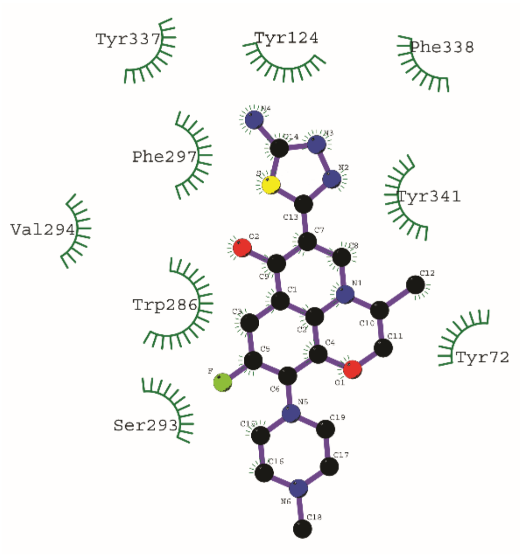

Figure. S2. Docking complex of 3a.

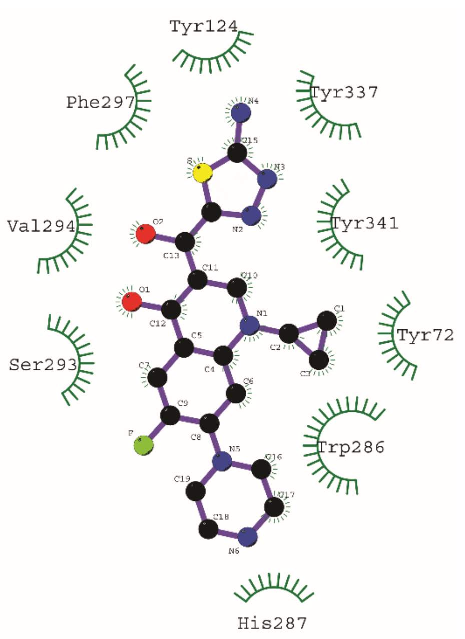

Figure S3. Docking complex of 3b.

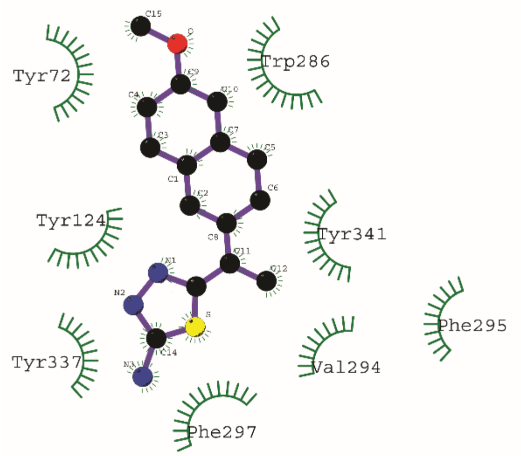

Figure S4. Docking complex of 3c.

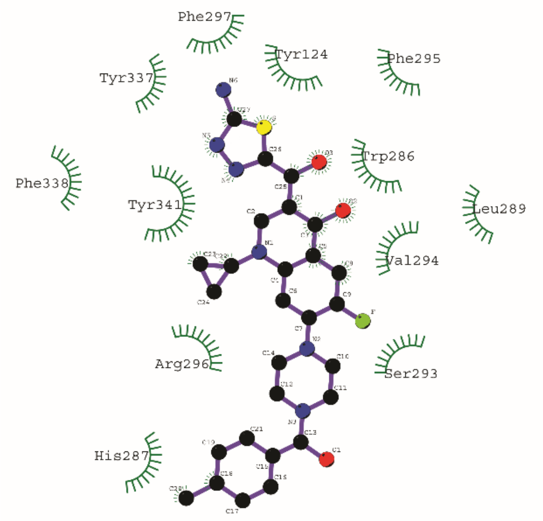

Figure S5. Docking complex of 3d.

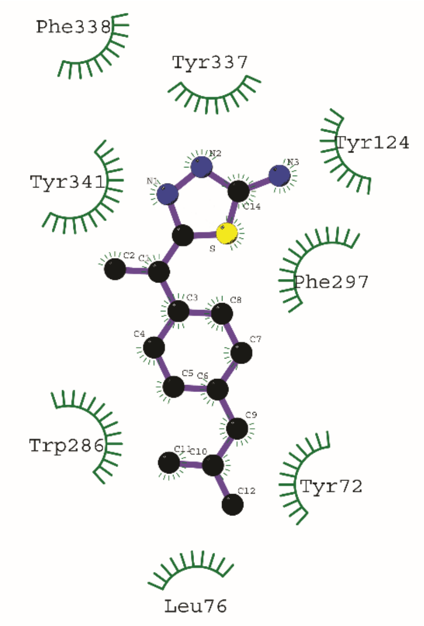

Figure S6. Docking complex of 3e.

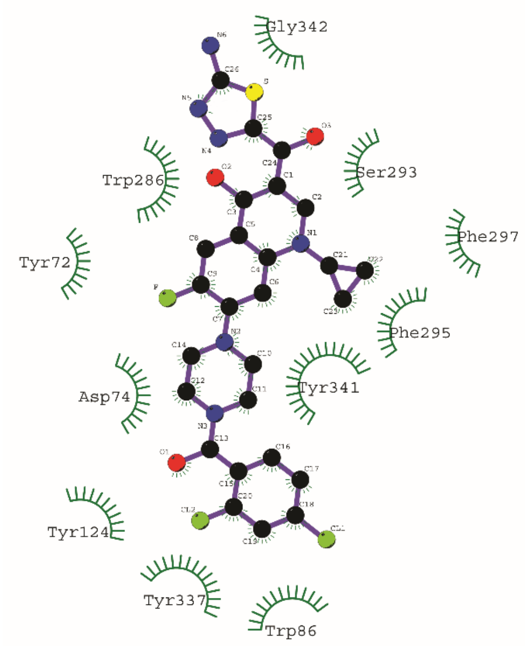

Figure S7. Docking complex of 3g.

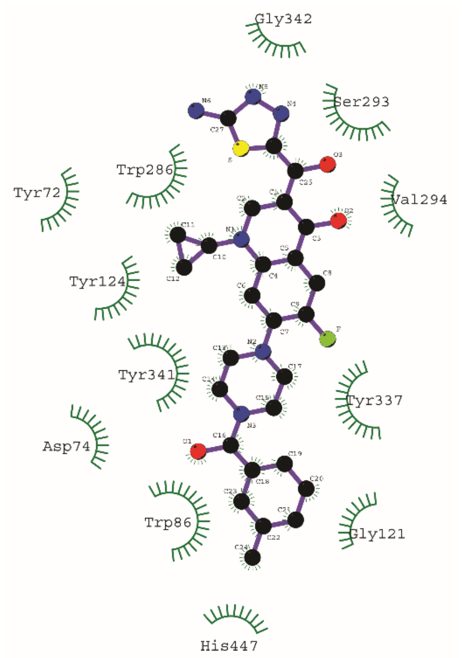

Figure S8. Docking complex of 3h.

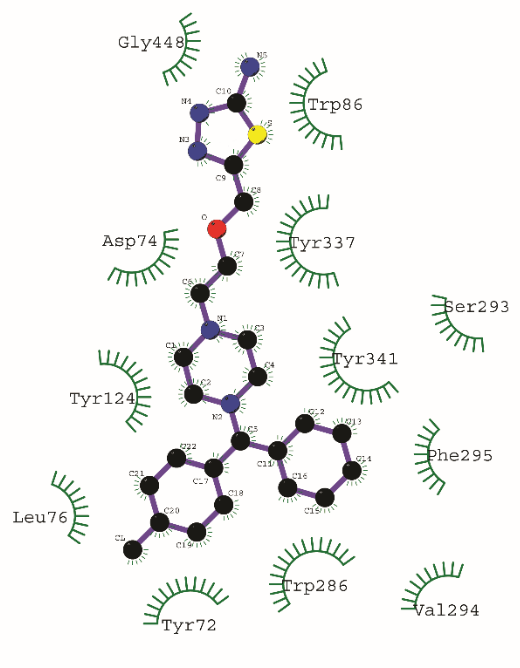

Figure S9. Docking complex of 3i.

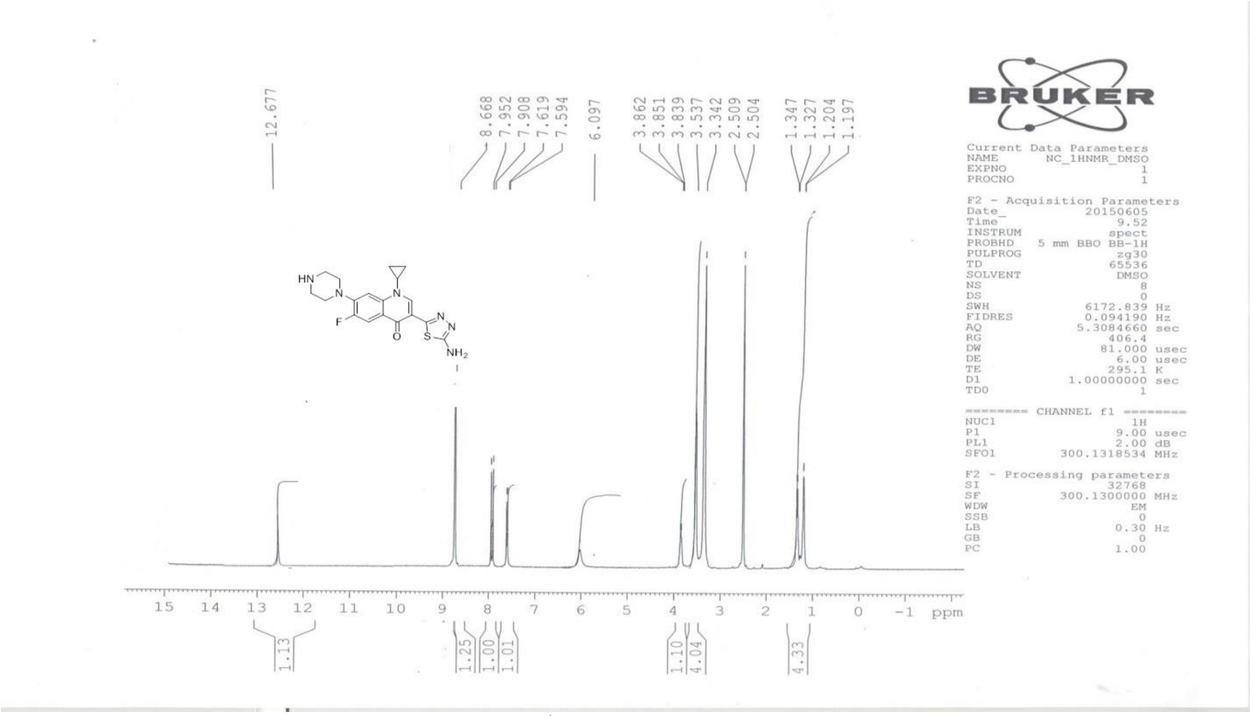

Figure S10. ^{1}H-NMR spectrum of **3a.**

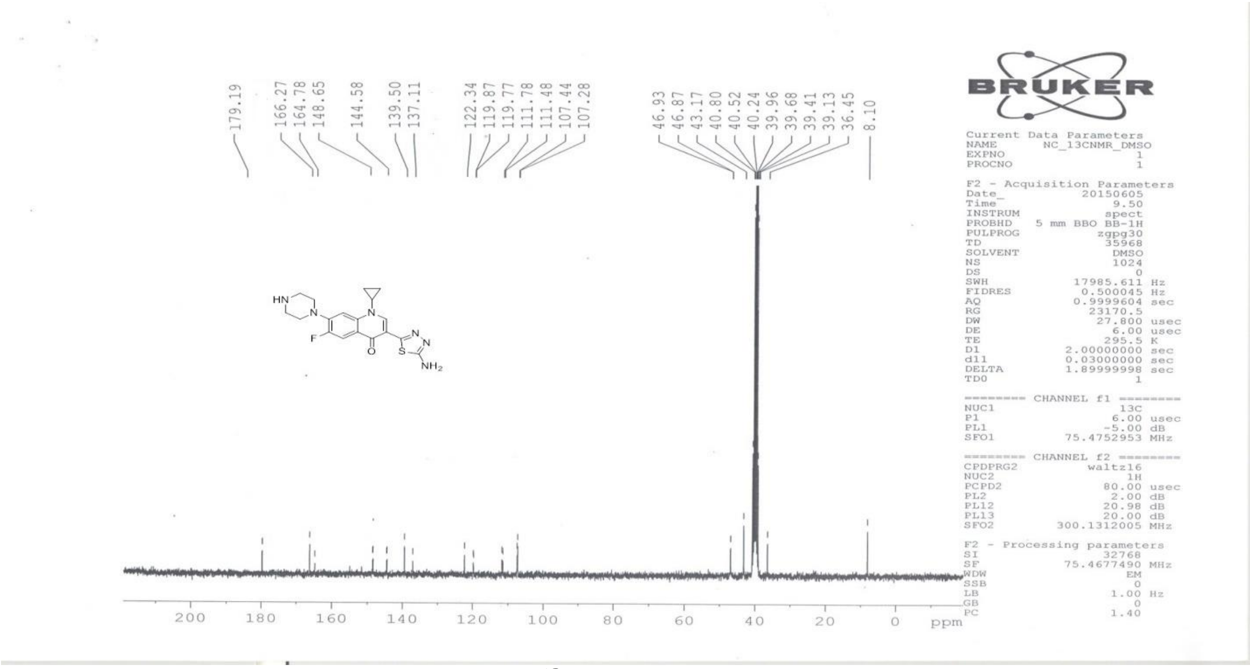

Figure S11. ^{13}C-NMR spectrum of **3a.**

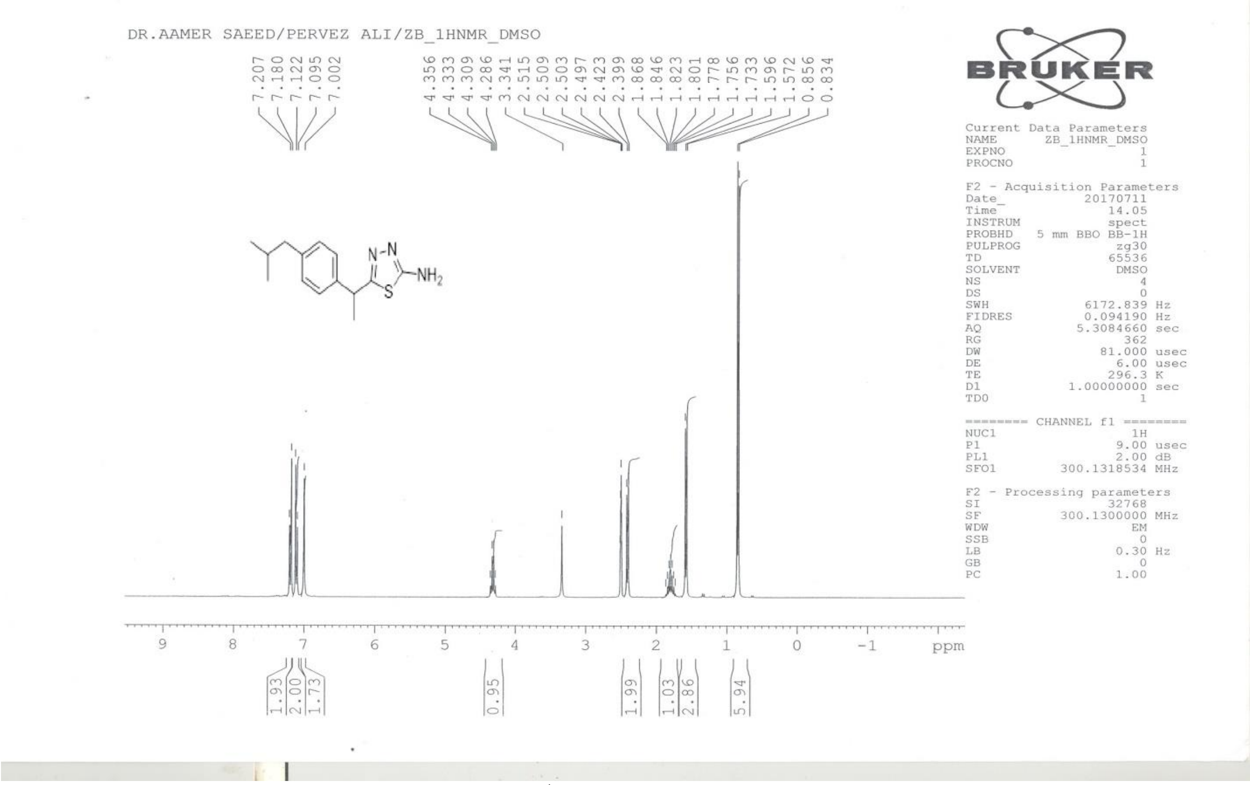

Figure S12. ^{1}H-NMR spectrum of **3d.**

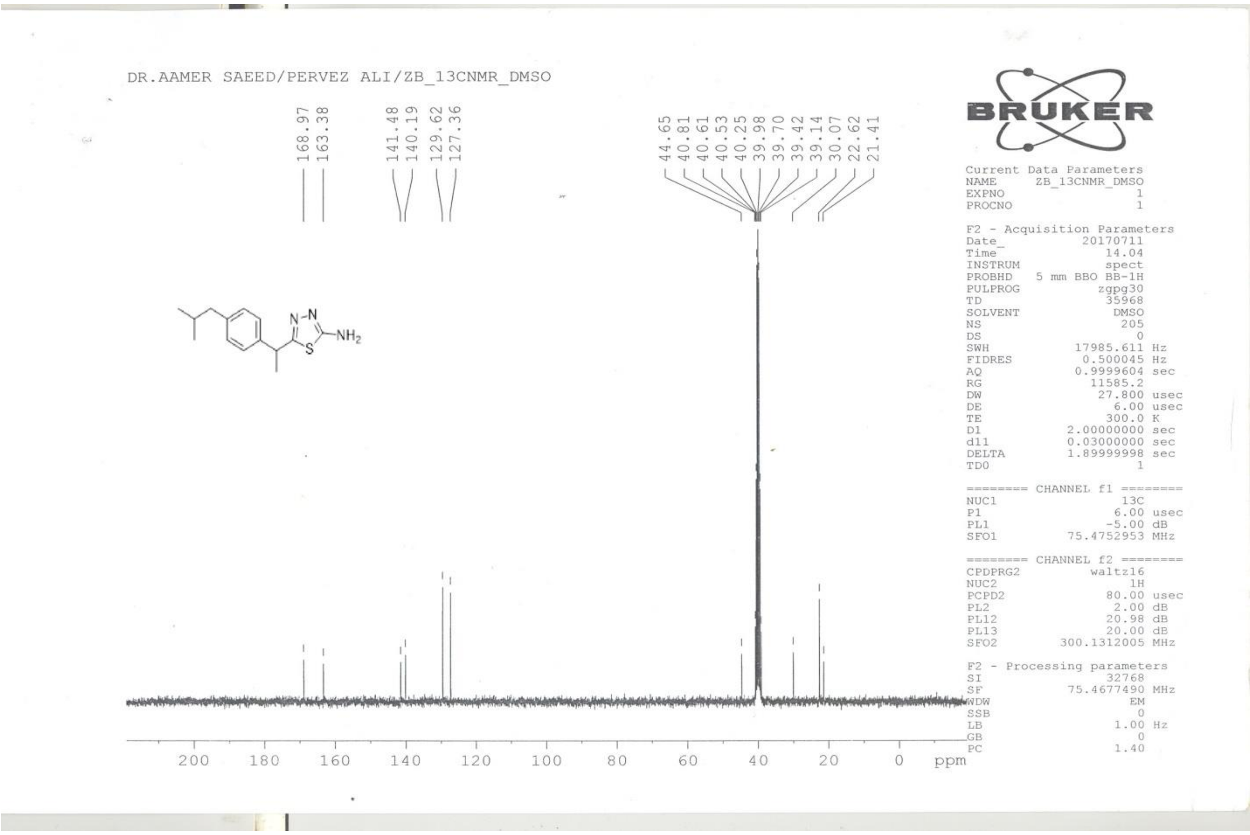

Figure S13. ^{13}C-NMR spectrum of 3d.
